# Risk factors associated with disease aggravation among 126 hospitalized patients with COVID-19 in different places in China: A retrospective observational study

**DOI:** 10.1097/MD.0000000000022971

**Published:** 2020-11-06

**Authors:** Shuai Shao, Zhiling Zhao, Feng Wang, Dandan Chang, Yong Liu, Shi Liu, Xiaoguang Xu, Xuyan Li, Chunguo Jiang, Ziren Tang

**Affiliations:** a Department of Respiratory and Critical Care Medicine, Beijing Chaoyang Hospital, Capital Medical University; b Beijing Institute of Respiratory Medicine; c Beijing Engineering Research Center for Diagnosis and Treatment of Respiratory and Critical Care Medicine; d Biomedical Engineering School, Beijing Key Laboratory of Fundamental Research on Biomechanics in Clinical Application, Capital Medical University, Beijing; e Department of Gastroenterology, Union Hospital, Tongji Medical College, Huazhong University of Science and Technology, Wuhan, Hubei Province; f Department of Respiratory and Critical Care Medicine, Zhoukou Central Hospital, Zhoukou, Henan Province; g Department of Respiratory and Critical Care Medicine, Affiliated Hospital of Jilin Medical University, Jilin; h Department of Emergency Medicine, Beijing Chaoyang Hospital, Capital Medical University, Beijing, China.

**Keywords:** coronavirus disease 2019, risk factor, severe acute respiratory syndrome, severe type

## Abstract

Coronavirus disease 2019 (COVID-19) has rapidly spread on a global scale. Therefore, it is urgent to identify risk factors that could be associated with severe type of COVID-19 from common type.

For this retrospective study, we recruited patients with COVID-19 in Wuhan and Zhoukou. Patients were classified into a severe group and common group based on guidelines after admission. Clinical manifestations and laboratory tests were compared, and univariate binary logistic regression and multivariate regression analyses were applied to assess potential risk factors.

A total of 126 patients were recruited from January 23 to March 23, 2020. Ninety cases were identified as the common type and 36 as the severe type. The average age in the severe group was significantly older than that in the common group (*P* = .008). Patients with severe COVID-19 exhibited higher proportions of dyspnea (*P* = .001), weakness (*P* = .023), and diarrhea (*P* = .046). Moreover, there were more patients with hypertension (*P* = .01) or coinfection (*P* = .001) in the severe group than in the common group. Additionally, severe COVID-19 was associated with increased neutrophil counts (*P* < .001), C-reactive protein (*P* < .001), procalcitonin (*P* = .024) and decreased lymphocyte counts (*P* = .001), hemoglobin (*P* < .001), total protein (TP) (*P* < .001), and albumin (ALB) (*P* < .001). Based on logistic regression analysis, dyspnea (*P* < .001), TP (*P* = .042), and ALB (*P* = .003) were independent risk factors for severe disease.

Patients with lower TP, ALB, and dyspnea should be carefully monitored, and early intervention should be implemented to prevent the development of severe disease.

**Highlights**

- Severe type of COVID-19 patients with hypertension need to be concerned.
- Ground-glass opacity and consolidation were most common in computed tomography.
- Dyspnea, lower total protein, and albumin were the risk factors of patients with severe SARS-CoV2 infection.

## 1 Introduction

Novel coronavirus disease 2019 (COVID-19) caused by severe acute respiratory syndrome coronavirus 2 (SARS-CoV-2) has been declared a pandemic, rapidly spreading to numerous countries such as China,^[1,2]^ Italy,^[3]^ America,^[4]^ and Canada.^[5]^ SARS-CoV-2 is a human coronavirus of the beta coronavirus genus that can enter host cells via angiotensin-converting enzyme 2.^[6]^

As of September, 2020, more than 17 million people have been diagnosed with COVID-19.^[7]^ Although COVID-19 has a lower rate of mortality than severe acute respiratory syndrome and middle east respiratory syndrome (10% and 37%, respectively),^[8,9]^ the intensive care unit mortality rate is almost 26%,^[10]^ and the mortality is as high as 88.1% among those who needed mechanical ventilation support.^[11]^ At present, researchers are searching for an effective treatment for patients with COVID-19, especially for those with faster and more severe disease progression. Ironically, even after a decade of study on coronaviruses, there are no vaccines or antiviral drugs that have been proven to effectively treat infection by these viruses. Indeed, months or even a year may be needed to develop a specific vaccine. This situation highlights an urgent need to identify risk factors that effectively indicate disease aggravation to help clinicians make decisions in a rapid and precise manner. Multiple studies to date have focused on describing the epidemiological features and clinical characteristics of patients with COVID-19 in detail.^[12–15]^ However, the prediction model for the diagnosis of disease severity and disease prognosis remains uncertain. Here, we construct a comprehensive assessment of common and severe cases of confirmed COVID-19 to explore potential risk factors for disease severity.

## 2 Methods

### 2.1 Data source

This study is a multicenter, retrospective study that recruited 126 patients aged 19 to 91 years old with confirmed COVID-19 pneumonia from January 23 and March 3, 2020. Patients data were collected from Union Hospital, Tongji medical college, Huazhong university of science and technology (Wuhan, Hubei Province of China), where patients were local ones and Zhoukou Central Hospital, where most patients where imported ones or second generation cases.

This study was approved by the Institutional Review Board of Wuhan Union Hospital (project approval number 2020–0127). Informed consent from each patient was waived since we collected and analyzed all of the data from each patient according to the policy for public-health-outbreak investigation of emerging infectious diseases issued by the National Health Commission of the People's Republic of China.

### 2.2 Data collection

Demographic data were collected by face-to-face interviews or telephone calls to patients or relatives, if available. The demographic and clinical data collected included the following: demographic characteristics (age and sex), underlying diseases, comorbidities, clinical symptoms, signs (body temperature, heart rate, respiratory frequency, and blood pressure), laboratory tests (blood routine test, arterial blood gas analysis, and blood chemistry), and images of the lung (chest CT). Treatment and complications were also recorded within 3 days after admission, and the results were extracted by our investigators.

Throat swab samples were obtained from all of patients, and COVID-19 pneumonia was diagnosed based on SARS-CoV-2 nucleic acid RT-PCR positivity of throat swab samples. The classification of common or severe disease was in accordance with the guidelines for diagnosis and management of COVID-19 issued by the National Health Commission of China 8^th^ edition.^[16]^ The criteria for the common type were as follows: fever and respiratory symptoms, with chest imaging abnormalities. The criteria of the severe type were as follows: respiratory rate ≥ 30/min; percutaneous oxygen saturation (SpO_2_) ≤ 93% while breathing ambient air; ratio of the partial pressure of oxygen (PaO_2_) to the fraction of inspired oxygen (FiO_2_) ≤ 300 mm Hg; chest imaging indicating the progression of lung lesions by more than 50% within 24 to 48 hours.

A trained team of investigators and medical students accessed the electronic medical records system after receiving approval from the hospitals to collect relevant data. All of the patient data were cross-checked for consistency before the final assessment, and all raw data were initially stored and evaluated by professional statisticians.

### 2.3 Statistical analysis

Data analysis was performed using SPSS 25.0 (IBM Corp., Armonk, NY) software. Continuous variables with a normal distribution are presented as the mean ± standard deviation; those with a nonnormal distribution are presented as the median. Categorical variables are summarized using frequencies and percentages. Independent group *t* tests were applied if the data were normally distributed; otherwise, the Mann–Whitney *U* test was used. Categorical variables were assessed by using the χ^2^ test or Fisher exact test when the sample size was small. Univariable binary logistic regression analyses were applied to explore clinical signs, symptoms, or laboratory findings with significant differences. Variables with a *P* value < .05 in the univariate analysis were entered into multivariate logistic regression analysis to identify independent risk factors associated with severe COVID-19. All *P* values less than.05 were considered statistically significant.

## 3 Results

### 3.1 Baseline characteristics

This retrospective study initially included 94 patients with common COVID-19 and 39 patients with severe COVID-19. Four patients with common COVID-19 and 3 patients with severe COVID-19 were excluded due to insufficient data on admission, leaving 126 patients for further analysis. The median age in the severe group was significantly greater than that in the common group (46.66 ± 14.81 years versus 58.14 ± 13.96 years, *P* = .008). The proportion of males was not significantly different between these 2 groups (*P* = .865). In terms of comorbidities, severe disease was associated with more comorbidities, such as coinfection (32 (35.6%) versus 24 (66.7%), *P* = .001) or hypertension (12 (13.3%) versus 12 (33.3%), *P* = .01). More details are presented in Table 1.

**Table 1:** Demographic characteristics of common patients and severe patients with coronavirus disease 2019.

Variables	Common group (N = 90)	Sever group (N = 36)	*P*
Age (yr)^∗^	46.66 ± 14.81	58.14 ± 13.96	.008
Gender Male, N (%)^†^	49 (54%)	19 (51%)	.865
Comorbidities, N (%)^†^	37 (41.1%)	17 (47.2%)	.531
Hypertension^†^	12 (13.3%)	12 (33.3%)	.01
Asthma^†^	1 (1.1%)	0	1.000
Diabetes^†^	7 (7.8%)	6 (16.7%)	.138
Malignancy^†^	2 (2.2%)	1 (2.8%)	1.000
Cardiovascular disease^†^	4 (4.4%)	2 (5.6%)	1.000
COPD^†^	0	2 (5.6%)	.080
Others^†^	11 (12.2%)	3 (8.3%)	.754
Co-infection, N (%)^†^	32 (35.6%)	24 (66.7%)	.001

### 3.2 Clinical manifestations

The basic clinical information of signs and symptoms in the common and severe groups are shown in detail in Table 2. The proportions of dyspnea (*P* < .001), weakness (*P* = .023), and diarrhea (*P* = .046) were significantly higher in the severe group than in the common group. Furthermore, the days from symptom onset to admission (5.00 (2.00, 10.50) days versus 9.50 (5.00, 12.00) days, *P* = .032), days from disease onset to improvement (8.50 (6.00, 13.25) days versus 16.00 (11.25, 20.75) days, *P* < .001), and duration of fever (7.00 (4.00, 11.00) days versus 12.00 (8.00, 15.00) days, *P* = .001) were much longer in the severe group than in the common group. Nine patients (10%) in the common group developed dyspnea, and the median time for dyspnea development was 7 days (7.00 (2.50, 13.50)). Thirty-four patients in the severe group had dyspnea, accounting for 94% of the severe cases, with a median time to develop dyspnea of 5 days (5.00 (3.00, 8.00)). In addition, severe disease was associated with a higher respiratory rate (21.00 (20.00, 22.00) beats/min versus 26.00 (23.25, 28.00) beats/min, *P* < .001).

**Table 2:** Clinical symptoms and signs between the common and severe groups with coronavirus disease 2019.

Variables	Common group (N = 90)	Sever group (N = 36)	*P*
D from symptom onset to admission (d)^∗^	5.00 (2.00, 10.50)	9.50 (5.00, 12.00)	.032
D from disease onset to improvement (d)^∗^	8.50 (6.00, 13.25)	16.00 (11.25, 20.75)	.000
D from PCR negative (d)^∗^	18.00 (13.00, 24.00)	21.00 (16.75, 24.50)	.349
Positive throat swab	88 (97.8%)	29 (80.6%)	.001
Symptoms			
Fever, N (%)^†^	77 (85.8%)	34 (94.4%)	.277
Duration of fever (d)^∗^	7.00 (4.00, 11.00)	12.00 (8.00, 15.00)	.001
Maximum body temperature (°C)^∗^	38.20 (38.00, 38.60)	38.55 (38.00, 39.00)	.033
Cough, N (%)^†^	56 (62.2%)	28 (77.8%)	.094
Dyspnea, N (%)^†^	9 (10.0%)	34 (94.4%)	.001
Pharyngalgia, N (%)^†^	14 (15.6%)	5 (13.9%)	.813
Rhinorrhea, N (%)^†^	3 (3.3%)	3 (8.3%)	.467
Headache, N (%)^†^	7 (7.8%)	4 (11.1%)	.803
Nasal congestion, N (%)^†^	3 (3.3%)	4 (11.1%)	.197
Weakness, N (%)^†^	45 (50.0%)	26 (72.2%)	.023
Mental symptoms, N (%)^†^	1 (1.1%)	1 (2.8%)	1.000
Myalgia, N (%)^†^	13 (14.4%)	10 (27.8%)	.080
Chills, N (%)^†^	9 (10.0%)	1 (2.8%)	.322
Conjunctival congestion	0	0	
Diarrhea, N (%)^†^	5 (5.6%)	6 (16.7%)	.046
Nausea or vomiting, N (%)^†^	6 (6.7%)	4 (11.1%)	.639
Chest pain, N (%)^†^	4 (4.4%)	0 (0.0%)	.577
Respiration rate (beats/min)^∗^	21.00 (20.00, 22.00)	26.00 (23.25, 28.00)	.000

### 3.3 Laboratory tests

Patients with severe COVID-19 exhibited higher absolute counts of neutrophils (NEUs) (3.29 (2.46, 4.2) × 10^9^/L versus 5.32 (3.92, 7.86) × 10^9^/L, *P* < .001) and levels of C-reactive protein (CRP) (8.06 (5.12, 30.91) mg/L versus 34.30 (18.97, 67.79) mg/L, *P* < .001), blood urea nitrogen (3.94 (3.03, 5.01) mmol/L versus 5.21 (3.53, 6.67) mmol/L, *P* = .016), lactic dehydrogenase (197.00 (156.50, 247.00) U/L versus 283.50 (204.00, 394.75) U/L, *P* < .001), and procalcitonin (PCT) (0.04 (0.02, 0.08) ng/ml versus 0.06 (0.04, 0.15) ng/mL, *P* = .024). In addition, results at the time of admission revealed that severe COVID-19 was associated with lower lymphocyte counts (1.28 (0.87, 1.77) × 10^9^/L versus 0.82 (0.63, 1.37) × 10^9^/L, *P* = .001) and total protein (TP) (70.85 (65.45, 76.83) g/L versus 54.05 (60.95, 65.88) g/L, *P* < .001), albumin (ALB) (44.30 (38.33, 49.60) g/L versus 28.50 (26.30, 33.15) g/L, *P* < .001), and creatine kinase levels (65.00 (48.10, 98.75) U/L versus 39.50 (22.75, 116.00) U/L, *P* = .011) compared to common COVID-19. Although these results were statistically significant, most of the laboratory indicators were within the normal range or just slightly higher than the upper limit of the normal value. Patients in the common group had a higher proportion of positive throat swabs than patients in the severe group (88 (97.8%) versus 29 (80.6%), *P* = .001). More information is provided in Table 3.

**Table 3:** Laboratory test findings between the common and severe groups with coronavirus disease 2019.

Variables	Normal value range	Common group (N = 90)	Sever group (N = 36)	*P*
**WBC (×10**^9^**/L)**	3.5–9.5	5.56 (4.01, 7.32)	5.98 (4.45, 10.18)	.063
Neutrophil count **(**×10^9^/L**)**	1.8–6.3	3.29 (2.46, 4.2)	5.32 (3.92, 7.86)	.000
Lymphocyte count **(**×10^9^/L**)**	1.1–3.2	1.28 (0.87, 1.77)	0.82 (0.63, 1.37)	.001
Neutrophil %	40–75	62.95 (55.30, 71.80)	78.80 (69.50, 83.55)	.000
Lymphocyte %	20–50	2.85 (2.00, 24.85)	10.75 (4.10, 18.83)	.812
RBC **(**×10^12^/L**)**	4.3–5.8	4.83 (4.21, 5.39)	4.08 (3.52, 4.59)	.000
Hemoglobin (g/L)	130–175	144.50 (130.00, 157.75)	121.0 (107.50, 144.25)	.000
Platelet **(**×10^9^/L**)**	125–350	206.50 (171.50, 239.00)	211.50 (184.50, 233.25)	.527
CRP (mg/L)	0–5.0	8.06 (5.12, 30.91)	34.30 (18.97, 67.79)	.000
Potassium (mmol/L)	3.5–5.3	4.11 (3.87,4.36)	3.73 (3.37, 4.03)	.000
Sodium (mmol/L)	137–147	139.40 (137.20, 143.50)	138.85 (137.73, 142.53)	.464
Total protein (g/L)	65–85	70.85 (65.45, 76.83)	54.05 (60.95, 65.88)	.000
Albumin (g/L)	40–55	44.30 (38.33, 49.60)	28.50 (26.30, 33.15)	.000
ALT (U/L)	9–50	28.50 (18.68, 48.70)	34.50 (22.75, 53.25)	.093
AST (U/L)	15–40	23.80 (18.75, 34.25)	28.50 (22.00, 49.50)	.077
Creatinine (umol/L)	57–97	64.70 (54.00, 73.90)	60.25 (53.93, 79.33)	.712
Bun (mmol/L)	2.9–7.5	3.94 (3.03, 5.01)	5.21 (3.53, 6.67)	.016
LDH (U/L)	120–250	197.00 (156.50, 247.00)	283.50 (204.00, 394.75)	.000
CK (U/L)	50–310	65.00 (48.10, 98.75)	39.50 (22.75, 116.00)	.011
CKMB (ng/mL)	0–5.0	10.10 (6.23, 16.25)	10.50 (6.00, 17.00)	.720
Tni (ng/mL)	0–0.04	0.31 (0.01, 2.73)	1.75 (0.01, 3.40)	.669
ESR (mm/h)	2–15	13.50 (11.00, 44.00)	11.50 (6.00, 48.75)	.214
Procalcitonin (ng/mL)	0–0.05	0.04 (0.02, 0.08)	0.06 (0.04, 0.15)	.024

### 3.4 Radiological tests

**Figure 1:**
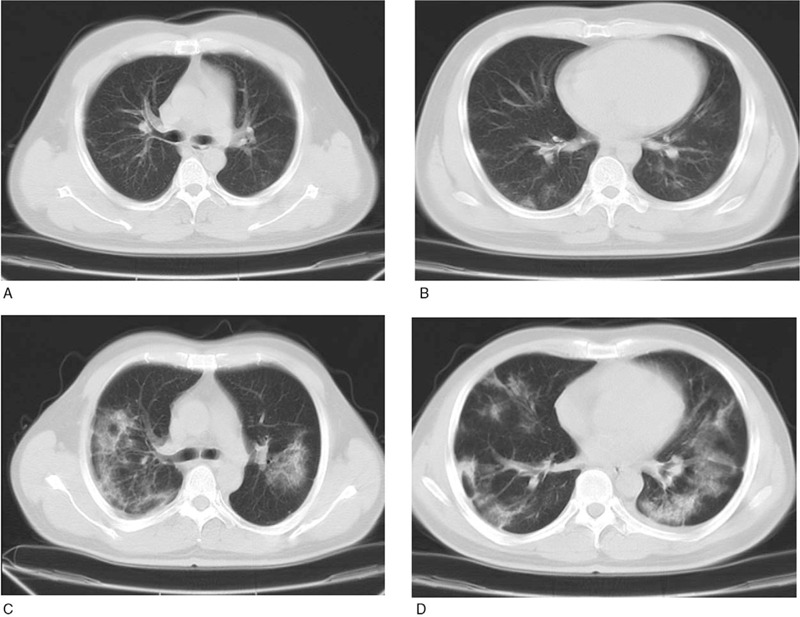
Imaging characteristics of chest computed tomography from common type of coronavirus disease 2019 (COVID-19) and severe type patients. A and B, 35-year-old male with common type COVID-19 patient exhibited multiple ground-glass opacities in both lungs. C and D, 49-year-old male with severe type COVID-19 exhibited diffuse ground-glass opacities and consolidation distributed in multiple lobes and segments.

All patients received a chest computed tomography CT scan within 3 days after admission. According to the results, 52 (74.3%) patients in the common group and 17 (81.0%) in the severe group had ground-glass opacities, and 21 (27.6%) patients in the common group and 2 (9.1%) in the severe group had patchy shadows. Consolidation was observed in 23 (31.9%) patients in the common group and 11 (50.0%) in the severe group. However, plural effusion was only detected in 3 patients, demonstrating a less common manifestation (Fig. 1).

### 3.5 Treatment regimen

In terms of treatment, more patients in the severe group were treated with antiviral agents, such as interferon (53 (58.9%) versus 10 (28.6%), *P* = .002), lopinavir/ritonavir (56 (62.2%) versus 11 (31.4), *P* = .002), and arbidol hydrochloride capsule (33 (36.7%) versus 22 (62.9%), *P* = .008) compared with those in the common group. In addition, more patients in the severe group received glucocorticoid therapy (19 (21.3%) versus 17 (50.0%), *P* = .002) and immunoglobulin therapy (9 (10.1%) versus 8 (25.0%), *P* = .038). Regarding adverse drug reactions between these 2 groups, 33 (37.1%) patients in the common group and 9 (29.0%) patients in severe group exhibited varied adverse drug reactions, but there was no significant difference between the groups. More details are presented in Table 4.

**Table 4:** Treatment regimen between the common and severe groups with coronavirus disease 2019.

Treatment regimen	Common group (N = 90)	Sever group (N = 36)	*P*
Lianhuaqingwen, N (%)	25 (27.8%)	4 (11.8%)	.101
Oseltamivir, N (%)	2 (2.2%)	0	1.000
Vidarabine, N (%)	3 (3.3%)	2 (5.9%)	.895
Interferon, N (%)	53 (58.9%)	10 (28.6%)	.002
Lopinavir/ritonavir, N (%)	56 (62.2%)	11 (31.4)	.002
Ribavirin, N (%)	28 (31.1%)	9 (25.7%)	.553
Arbidol Hydrochloride, N (%)	33 (36.7%)	22 (62.9%)	.008
Chloroquine, N (%)	1 (1.1%)	1 (2.9%)	1.000
Xuebijing injection, N (%)	37 (41.6%)	11 (31.4%)	.297
Glucocorticoids, N (%)	19 (21.3%)	17 (50.0%)	.002
Anti-bacteria therapy, N (%)	56 (64.4%)	25 (73.5%)	.336
Immunoglobulin, N (%)	9 (10.1%)	8 (25.0%)	.038
Adverse drug reactions, N (%)	33 (37.1%)	9 (29.0%)	.419

### 3.6 Multivariate analysis

**Figure 2:**
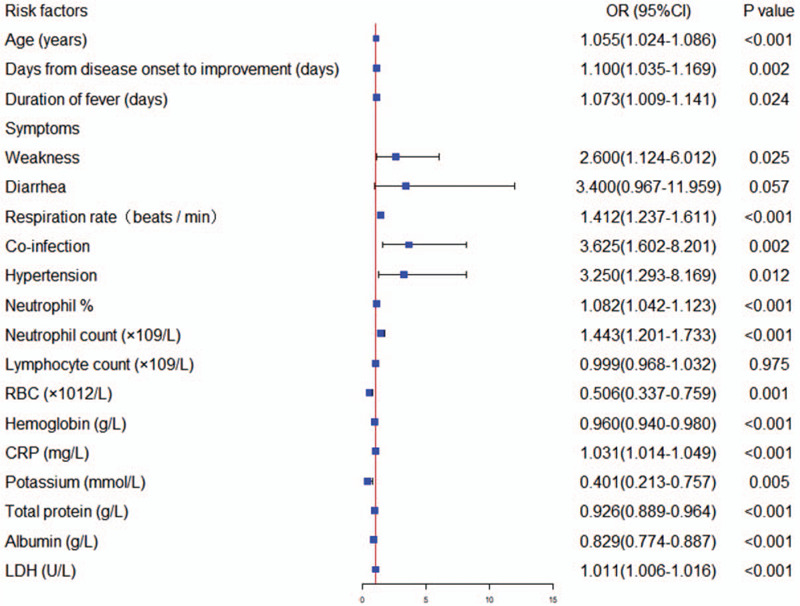
Multivariate regression analysis for specific risk factors for coronavirus disease 2019 of severe type from common type. Plots reporting variables independently associated with the risk for severe type of coronavirus disease 2019 in the final model, with their 95% confidence intervals. COVID-19 = corona virus disease 2019, RBC = red blood cell, CRP = C-reactive protein, LDH = lactic dehydrogenase, BUN = blood urea nitrogen, CK = creatine kinase, OR = odds ratio, CI = confidence interval.

Variables with a *P* value < .05 in the univariate analysis were entered into multivariate logistic regression analysis. Compared with the common type, patients with severe disease were more likely to have dyspnea (OR 1732.85; 95% CI [41.40 - 72526.21]; *P* < .001), lower TP (OR 0.88; 95% CI 0.78–0.99; *P* = .042), and lower ALB (OR 0.79; 95% CI [0.67–0.92]; *P* = .003) (Table 5 and Fig. 2). We did not perform Cox regression or Kaplan–Meier survival curve analysis because only 1 patient in the severe group died, with no deaths in the common group.

**Table 5:** Univariable and multivariate analysis of independent risk factors for differentiating coronavirus disease 2019 of severe type from common type.

	Univariate analysis			Multivariate analysis		
		
	OR	95% CI	*P*	OR	95% CI	*P*
Age (yr)	1.055	1.024–1.086	.000			
D from disease onset to improvement (d)	1.100	1.035–1.169	.002			
Duration of fever (d)	1.073	1.009–1.141	.024			
Symptoms						
Dyspnea	153.00	31.399–745.529	.000	1732.85	41.40, 72526.21	<.001
Weakness	2.600	1.124–6.012	.025			
Respiration rate (beats/min)	1.412	1.237–1.611	.000			
Co-infection	3.625	1.602–8.201	.002			
Hypertension	3.250	1.293–8.169	.012			
Neutrophil %	1.082	1.042–1.123	.000			
Neutrophil count **(**×10^9^/L**)**	1.443	1.201–1.733	.000			
RBC **(**×10^12^/L**)**	0.506	0.337–0.759	.001			
Hemoglobin (g/L)	0.960	0.940–0.980	.000			
CRP (mg/L)	1.031	1.014–1.049	.000			
Potassium (mmol/L)	0.401	0.213–0.757	.005			
Total protein (g/L)	0.926	0.889–0.964	.000	0.88	0.78–0.99	.042
Albumin (g/L)	0.829	0.774–0.887	.000	0.79	0.67–0. 92	.003
LDH (U/L)	1.011	1.006–1.016	.000			
Procalcitonin (ng/mL)	123.157	1.944–7800.828	.023			

## 4 Discussion

This study comprehensively provides data underlying the demographic, clinical, laboratory, and radiological characteristics as well as the treatment of 126 patients diagnosed with COVID-19. We found dyspnea, lower TP, and lower ALB to be risk factors for disease aggravation in patients with COVID-19.

ALB, hemoglobin, total cholesterol, and TP were explored in a previous meta-analysis as useful markers of adult malnutrition.^[17]^ Adult malnutrition, especially protein-energy malnutrition, always causes defects in the human immune system and disease progression. Additionally, prior studies have suggested that protein-energy malnutrition is associated with significant impairment of several aspects of immunity, such as secretory immunoglobulin A antibody and cytokine production, cell-mediated immunity, phagocyte function, and complement.^[18,19]^ Fever, cough and sputum are common clinical manifestations of patients with COVID-19.^[1]^ Dyspnea suggests poor lung function and lack of oxygen. Therefore, when patients are found to have low ALB or TP levels or difficulty breathing, it is necessary for clinicians to be alerted to further deterioration of the patient's condition.

Previous studies have reported that older age is one of the important risk factors for death in COVID-19.^[20,21]^ However, in this study, we did not find an association between age and disease aggravation. This inconsistency might be because the patients recruited in our study were relatively young, with an average age of 49.9 years. In addition, the primary outcome of our study was risk factors for disease aggravation rather than for death. Although age was not a risk factor to predict aggravation of disease through logistic regression in this study, the patients in the severe group were older than those in the common group. Moreover, hypertension was not detected as a risk factor in our study, which is different from previous studies.^[22]^ Several studies have reported that angiotensin-converting enzyme 2 (ACE2) plays a role as a gateway for SARS-CoV-2 and other coronaviruses,^[23]^ and ACE inhibitors (ACEIs) and angiotensin receptor blockers (ARBs) have been speculated to increase damage to the lung, which might enhance expression of ACE2 and help SARS-CoV-2 enter the host cell.^[24]^ However, to date, no research has proven this hypothesis. Moreover, there is no definitive evidence to confirm that hypertension is associated with increased expression of ACE2 and whether this expression might contribute to poor outcomes in patients with COVID-19.^[25,26]^ At the same time, a study did report that ACEI did not inhibit ACE2, making the harmful effect of hypertension unlikely.^[27]^ Although ARBs have been reported to upregulate ACE2 in animal models,^[28,29]^ it is unclear whether these findings could translate into clinical conditions (such as patients with COVID-19). Furthermore, the use of ACEIs and ARBs in China is relatively low; in 1 study, renin-angiotensin system inhibitors were reported to be used in 25% to 30% of treated patients.^[30]^ These factors might be the reasons that hypertension was not a risk factor in our study. Overall, more prospective studies are warranted to confirm the association between hypertension and disease progression in COVID-19 patients.

Regarding treatment between the 2 groups, more patients in the severe group received lopinavir/ritonavir, arbidol hydrochloride capsule, and glucocorticoid therapy. To date, only dexamethasone has been indicated to decrease the mortality of patients with COVID-19.^[31]^ One study involving 199 patients with laboratory-confirmed SARS-CoV-2 infection showed that lopinavir/ritonavir offered no significant treatment effect compared with standard care.^[32]^ In addition, there have been no large-sample-size, high-quality studies to confirm the efficacy of arbidol, interferon, or immunoglobulin in patients in COVID-19.^[33–35]^ In our study, the different treatments between the 2 groups were not found to be a risk factor. Though there are no other proven options for the treatment of COVID-19, the various treatment strategies between the common and severe groups might be confounding factors with regard to influencing disease aggravation and identifying other risk factors, which should be recognized.

Patients with severe COVID-19 always have lymphopenia and decreased numbers of CD4+ T cells, CD8+ T cells, B cells, and natural killer cells.^[1,36,37]^ Wynants et al performed a systematic review by constructing prediction models for COVID diagnosis and prognosis,^[38]^ showing that CRP and lactic dehydrogenase levels and lymphocyte counts were the most commonly mentioned predictors. Chen et al explored the relationship of longitudinal hematologic and immunologic variations between patients with different outcomes and disease severity^[39]^; the pooled results revealed that the counts of lymphocytes, T cell subsets, eosinophils, and platelets were remarkably decreased in the severe or critical/fatal group, and significant increases in NEU count, CRP, and PCT in the common/survival group. For COVID-19, dynamic changes in hematologic and immunologic markers, such as progressive decline in eosinophils, lymphocytes, and platelets and dynamic increases in NEUs, IL-6, PCT, D-dimer, and CRP, during hospitalization are indeed strong suggestions for progression of the disease. SARS-CoV-2 mainly attacks the lung, and diffuse alveolar damage is the predominant lung pathology.^[40]^ Postmortem autopsy has revealed that in addition to the lung, damage to the liver, spleen, kidney, and microvascular system occurs during SARS-CoV2 infection.^[41]^ Several case series have also shown that patients with COVID-19 usually have increased aspartate aminotransferase, alanine, cardiac troponin, or creatine kinase-MB levels, even though this trend was not obvious in our study.^[42–46]^ Roosecelis et al published an article about the clinicopathologic, immunohistochemical, and electron microscopic discoveries in tissues from 8 fatal confirmed cases of SARS-CoV-2 infection in the America^[40]^ and found SARS-CoV-2 in the lung tissue by immunohistochemistry, with no evidence detected in the heart, liver, kidney, spleen, or intestine. Therefore, organ damage to the heart and liver, among other organs, may not directly result from damage due to SARS-CoV-2 but due to overactivation of both innate and adaptive immune responses. Indeed, the uncontrolled immune response may cause harmful tissue damage to the body.^[47]^

Our study has several strengths. First, as our research is a multicenter study, the research is to some extent and representative, with abundant data. Our study also examined risk factors for disease progression and found implications for hypoproteinemia and dyspnea, which are particularly important, in the treatment of COVID-19 patients. At the same time, through analysis of laboratory and imaging indicators, we further detected differences in clinical characteristics between patients with common and severe COVID-19. There are also some limitations in our article. First, recall bias is difficult to avoid due to the study design, even though we collected all of the data as soon as possible after the patients were admitted. Second, data for some variables, such as laboratory findings, CT scans, and clinical courses, were missing, which might cause bias in the accuracy and reliability of our results. Third, the laboratory tests collected were all performed at admission or within 3 days after admission, and the lack of preadmission laboratory tests and laboratory tests in the process of hospitalization makes results in a lack of relevant experimental results for COVID-19 patients in the course of the disease. In addition, because multiple comparisons were carried out, and inflated type I error should be considered.^[48,49]^

## 5 Conclusion

Although most of the patients can recover during the pandemic period of COVID-19, there is still a high mortality rate in severe patients. In this paper, 126 patients with COVID-19 were reviewed systematically to analyze the risk factors related to disease severity. The results suggested that we should pay more attention to patients with hypertension and co-infection, and dyspnea, lower TP, and ALB were the risk factors of patients with severe SARS-CoV2 infection.

## Acknowledgments

We thank all colleagues who collected and computed medical information of the patients included in this study. We also thank all the doctors, nurses, and clinical scientists who worked in the hospital during the period of patient recruitment, as well as all patients and their families who were involved in the study.

## Author contributions

Feng Wang developed the initial idea of this study and collected the data. Zhiling Zhao and Dandan Chang performed statistical analysis. All authors have made their contributions to research design, interpretation of results, and ideas for writing articles. Shuai Shao and Zhiling Zhao drafted the article. Ziren Tang and Feng Wang reviewed this article and provided suggestion for it. All of the authors have carefully examined this manuscript and agreed with the ideas presented in the article.

**Conceptualization:** Shuai Shao, Feng Wang.

**Data curation:** Dandan Chang, Feng Wang, Shi Liu, Yong Liu, Ziren Tang.

**Formal analysis:** Dandan Chang.

**Funding acquisition:** Feng Wang.

**Investigation:** Zhiling Zhao, Dandan Chang, Feng Wang, Yong Liu, Xiaoguang Xu, Xuyan Li, Chunguo Jiang.

**Methodology:** Dandan Chang, Shi Liu.

**Resources:** Feng Wang.

**Software:** Dandan Chang.

**Supervision:** Zhiling Zhao, Feng Wang, Ziren Tang.

**Validation:** Shuai Shao, Zhiling Zhao, Feng Wang.

**Visualization:** Zhiling Zhao.

**Writing – original draft:** Shuai Shao.

**Writing – review & editing:** Shuai Shao.
